# Traumatic Brain Injury as Frequent Cause of Hypopituitarism and Growth Hormone Deficiency: Epidemiology, Diagnosis, and Treatment

**DOI:** 10.3389/fendo.2021.634415

**Published:** 2021-03-15

**Authors:** Valentina Gasco, Valeria Cambria, Fabio Bioletto, Ezio Ghigo, Silvia Grottoli

**Affiliations:** Division of Endocrinology, Diabetes and Metabolism, University of Turin, Turin, Italy

**Keywords:** traumatic brain injury, hypopituitarism, growth hormone deficiency, pituitary, brain damage

## Abstract

Traumatic brain injury (TBI)-related hypopituitarism has been recognized as a clinical entity for more than a century, with the first case being reported in 1918. However, during the 20^th^ century hypopituitarism was considered only a rare sequela of TBI. Since 2000 several studies strongly suggest that TBI-mediated pituitary hormones deficiency may be more frequent than previously thought. Growth hormone deficiency (GHD) is the most common abnormality, followed by hypogonadism, hypothyroidism, hypocortisolism, and diabetes insipidus. The pathophysiological mechanisms underlying pituitary damage in TBI patients include a primary injury that may lead to the direct trauma of the hypothalamus or pituitary gland; on the other hand, secondary injuries are mainly related to an interplay of a complex and ongoing cascade of specific molecular/biochemical events. The available data describe the importance of GHD after TBI and its influence in promoting neurocognitive and behavioral deficits. The poor outcomes that are seen with long standing GHD in post TBI patients could be improved by GH treatment, but to date literature data on the possible beneficial effects of GH replacement therapy in post-TBI GHD patients are currently scarce and fragmented. More studies are needed to further characterize this clinical syndrome with the purpose of establishing appropriate standards of care. The purpose of this review is to summarize the current state of knowledge about post-traumatic GH deficiency.

## Introduction

Traumatic brain injury (TBI) is one of the leading causes of disability and mortality affecting many people each year and resulting in a serious burden of devastating health consequences (1–3). The most common mechanism for TBI are falls, especially in older adults and very young children; motor vehicle accidents are instead the most frequent cause of TBI among young adults (1). TBI may lead to permanent or transient pituitary insufficiency (4, 5). The clinical picture presents a very large spectrum determined by the kind, number and severity of hormonal deficiency and could go from mild and non-specific complaints to life-threatening conditions. The reported prevalence of hypopituitarism is quite variable among the available studies (6–9). The pathophysiologic mechanisms underlying pituitary damage in TBI patients include a primary injury that may lead to direct trauma to the hypothalamus or pituitary gland, or to compressive effect from surrounding structures; secondary injuries, on the other hand, are mainly related to an interplay of a complex and ongoing cascade of specific molecular/biochemical events. The diagnosis of pituitary dysfunction is very challenging both due to the common occurrence of TBI, the subtle character of clinical manifestations, the variable course of the disease, as well as the lack of proper diagnostic algorithms. Growth hormone deficiency (GHD) is the most common abnormality, followed by hypogonadism, hypothyroidism, hypocortisolism, and diabetes insipidus (8, 9). The purpose of this review is to summarize the current state of knowledge about post-traumatic hypopituitarism, and especially about post-traumatic GHD. However, well-designed studies are needed to further investigate the pathophysiology, epidemiology, and timing of pituitary dysfunction after a TBI with the purpose of establishing appropriate standards of care.

## Epidemiology of TBI and TBI-Related Hypopituitarism

### Epidemiology of TBI

TBI is one of the leading causes of disability and mortality in western countries. It can be estimated that in the United States, every year there are around 2.5 million visits at the emergency department, 280,000 hospitalizations and 50,000 deaths related to TBI (1).

The highest rates of TBI are observed in older adults (≥75 years; 2232 per 100,000 population), followed by very young children (0 to 4 years; 1591 per 100,000) and young adults (15 to 24 years; 1081 per 100,000). There is a sex prevalence, with males showing a higher risk than females (959 per 100,000 vs 811 per 100,000). Falls are the most common mechanism for TBI, especially in older adults and very young children; motor vehicle accidents are instead the most frequent cause of TBI among young adults (1).

After the acute phase, TBI-survivors are often forced to deal with relevant and persistent long-term sequelae, with significant neurological and functional impairment. The prevalence of TBI-related long-term disability in the United States is estimated to affect between 3 and 6 million patients, i.e., 1% to 2% of the population (2, 3).

Apart from the individual sequelae, TBI clearly determines significant economic implications for society, related both to direct expenses for medical care and to indirect costs caused by injury-related work loss and disability (10). The former can be as high as 80,000 US dollars per person in the first year after trauma (11). The latter is more difficult to estimate, but it is likely to account for more than 80% of the total economic burden of TBI (10).

### Epidemiology of TBI-Related Hypopituitarism

TBI-related hypopituitarism has been recognized as a clinical entity for more than a century, with the first case being reported in 1918 (12). However, during the 20^th^ century it was considered only a rare sequela of TBI.

Most likely, hypopituitarism was under-recognized for such a long time for its generally subtle and nonspecific clinical features that also share a significant overlap with many of the somatic, psychiatric and neurological symptoms directly related to TBI. As a consequence, only patients with the frankest clinical pictures were probably identified as having a TBI-related hypopituitarism, leaving unrecognised the vast majority of TBI patients with some degree of pituitary deficits. This underestimation may probably have affected patient life expectancy and quality, as it happens to every patient living with unrecognised and untreated hypopituitarism (13).

The awareness of the critical relevance of hypopituitarism in TBI patients radically changed during the last two decades. The first two cornerstone studies that solidly proved that TBI-related hypopituitarism was a far more common sequela of head trauma than previously thought have been published in 2000 and 2001 (4, 5). Since then, several other studies of the endocrine function in patients after TBI have been published (6, 7, 14–40).

The reported prevalence of hypopituitarism is quite variable among the available studies, ranging from 1% (6) to 76% (7). However, this relevant heterogeneity should not surprise because the existing studies widely differ in many aspects, such as study design, age of patients, severity of trauma, time point of endocrinological evaluation and testing protocols for the diagnosis of the deficiency of the various pituitary axes.

Given these premises, an overall summary of the available evidence can be found in two major meta-analyses (8, 9). Pooled data show that the proportion of patients with some kind of pituitary disfunction can be estimated to be approximately 27.5-32.0% (8, 9). In most individuals only a single pituitary axis is affected (19.8-25.3%) (8, 9), while involvement of multiple pituitary axes is far less frequent (6.7-7.7%) (8, 9).

Furthermore, it has been consistently proved that not all pituitary axes are equally susceptible to TBI-mediated damage. The most sensitive ones appear to be GH and FSH/LH (12.4-22.1% and 10.2-12.5%, respectively) (8, 9); on the other hand, deficits of ACTH and TSH axes appear to be significantly less frequent (8.2-9.9% and 4.1-6.2%, respectively) (8, 9).

## Pathophysiology of TBI-Related Hypopituitarism

### Overview on the Pathophysiology of TBI

TBI is a heterogeneous disease. There are many ways to categorize the patients, both in terms of clinical severity and pathophysiological mechanism of injury.

Clinical severity is usually assessed by specific severity scores; the most commonly used is the Glasgow Coma Scale (GCS), which evaluates three neurological domains (eye opening, best verbal response, best motor response) and classifies TBI as mild (GCS 13-15), moderate (GCS 9-12) or severe (GCS ≤ 8) (41).

The pathophysiology of TBI is usually summarized into two separate categories: primary and secondary brain injury (42).

Primary brain injury occurs at the time of trauma, as a consequence of external mechanical forces transferred to intracranial content. The pathologic sequelae of primary brain injury include shearing of white matter tracts (also known as diffuse axonal injury), focal cerebral contusion/hemorrhages, and focal extra-axial hematomas/hemorrhages (i.e. epidural hematomas, subdural hematomas, subarachnoid hemorrhage and intraventricular hemorrhage) (42, 43).

Following this primary injury, extensive and lasting damage is sustained through a complex and ongoing cascade of events referred to as secondary brain injury. Pathogenesis is driven by complex, interacting mechanisms that include, among others, neurotransmitter-mediated excitotoxicity, secondary ischemia (from vasospasm or other secondary vascular injuries, such as focal microvascular occlusion), and inflammatory responses. As a final consequence, these mechanisms of injury lead to neuronal cell death, cerebral edema and increased intracranial pressure, which can further exacerbate brain damage (42, 44, 45).

### Pathophysiology of TBI-Related Hypopituitarism

From a general point of view, the pathophysiological mechanisms underlying pituitary damage in TBI-patients are broadly similar to those described for TBI itself.

**Figure 1:**
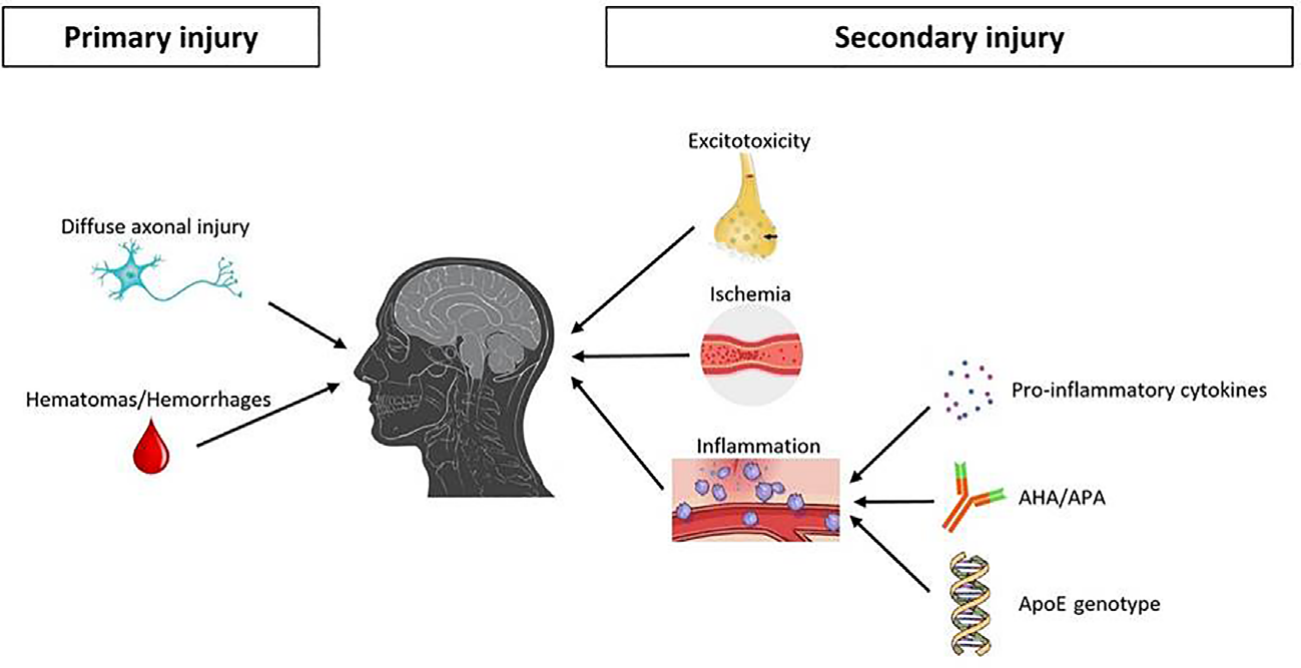
Main pathophysiological mechanisms underlying pituitary damage after TBI. TBI, traumatic brain injury; AHA, anti-hypothalamus antibodies; APA, anti-pituitary antibodies.

Primary injury may lead to direct trauma of the hypothalamus or pituitary gland, or to compressive effect from surrounding structures (42, 43); moreover, especially in case of skull base fracture, primary injury may determine pituitary stalk transection (46) (**Figure 1**).

On the other hand, secondary injuries are mainly related to an interplay of a complex and ongoing cascade of specific molecular/biochemical events (42, 44, 45) (**Figure 1**).

As already discussed, one of the three major mechanisms for secondary brain injury after head trauma is represented by excitotoxicity. Excitotoxicity is caused by the abnormal levels of excitatory neurotransmitters (mostly glutamate) that are uncontrollably released in patients with TBI. In fact, at high concentrations, these neurotransmitters act as excitotoxins, operating on ion channels and thus altering cell wall permeability with an unregulated electrolyte shift between intra- and extracellular spaces (47).

**Figure 2:**
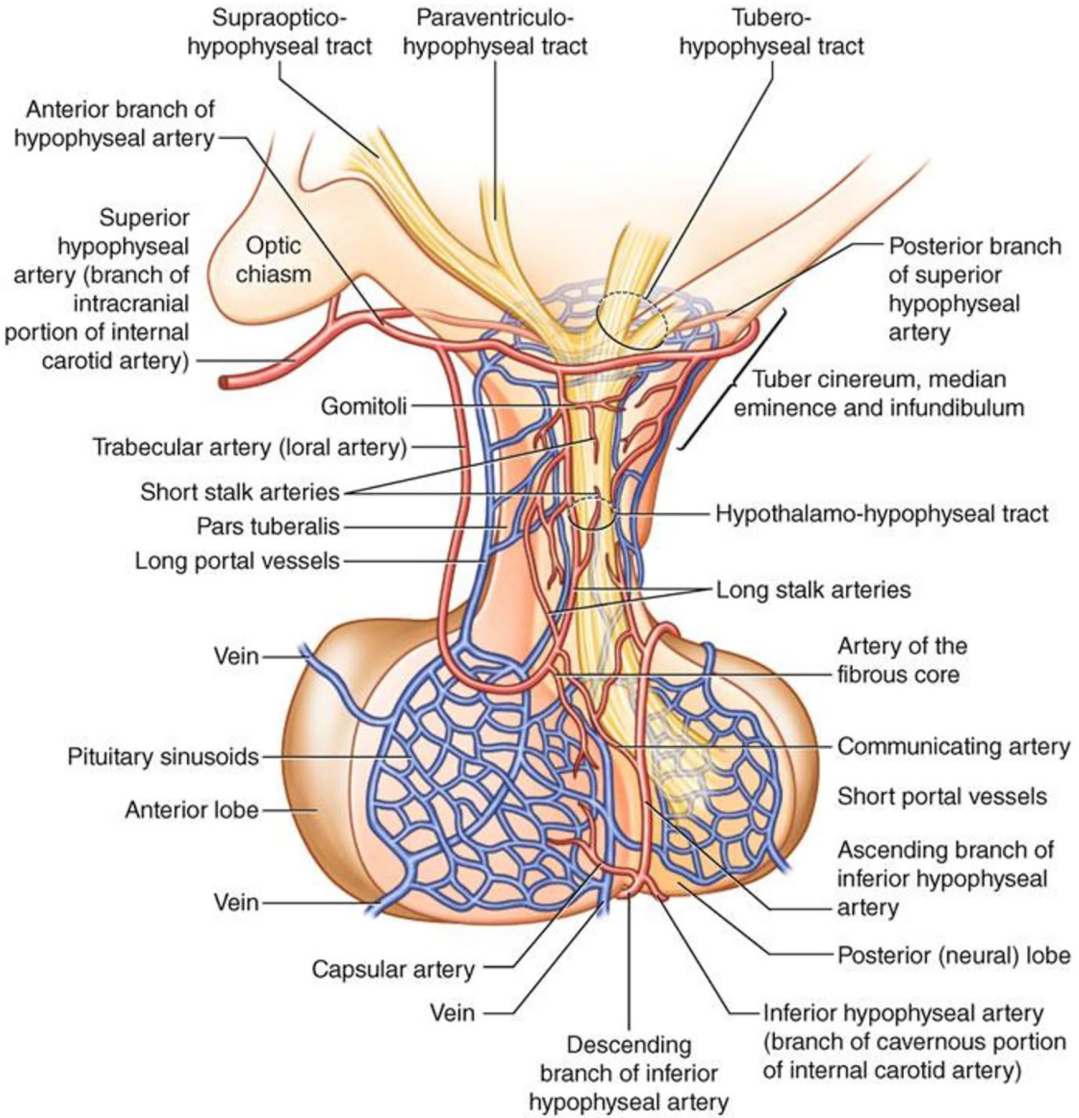
Anatomy and vascularization of the hypothalamus and pituitary gland [reproduced with permission from (51)].

**Figure 3:**
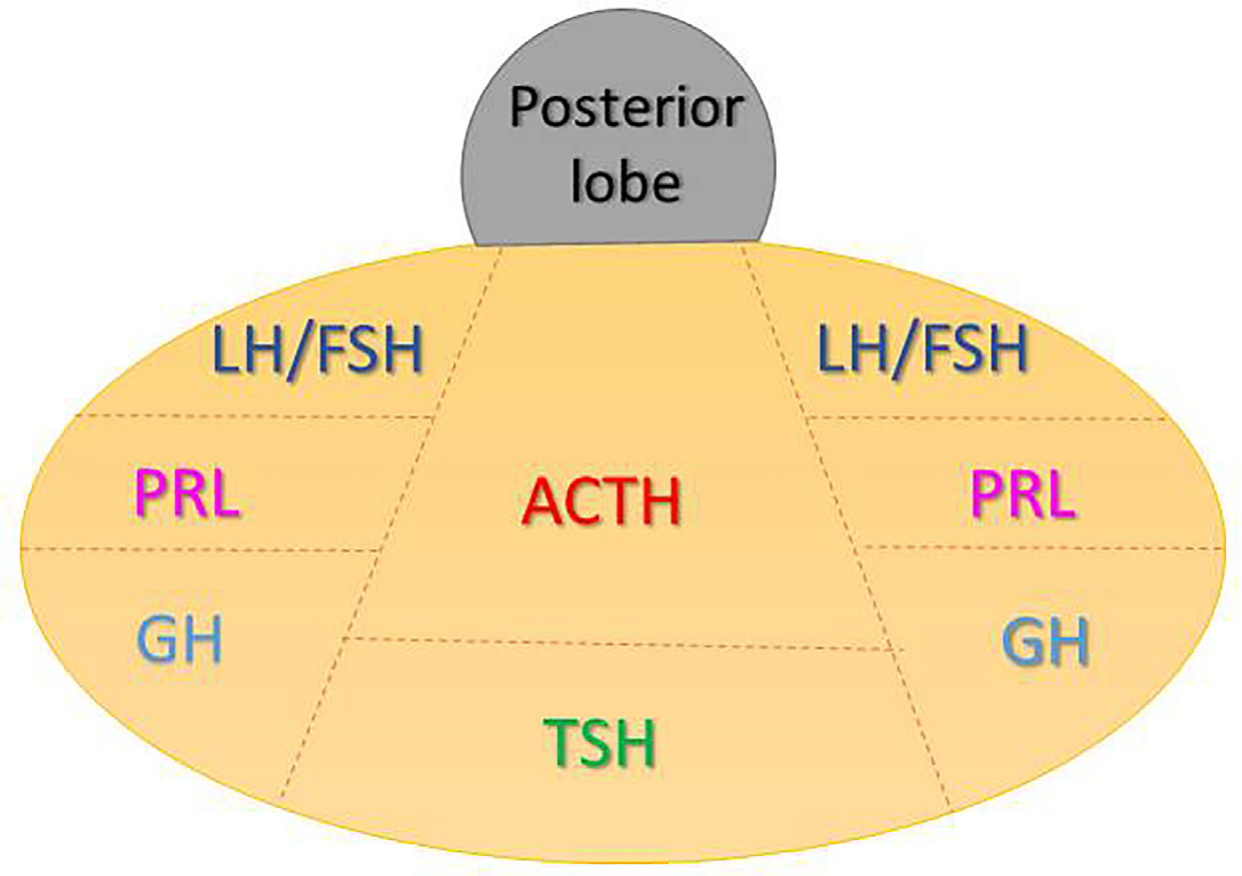
Distribution of anterior pituitary cell subtypes.

Another mechanism for secondary brain damage after TBI is represented by ischemia. Overall, the same pathophysiological events affecting brain are likely to underlie the pituitary-specific ischemic insult as well. However, some distinctive points related to the peculiar vascularization of the hypothalamic-pituitary area are still worth to be discussed. As known, the anterior pituitary receives its blood supply from the hypothalamic-hypophyseal portal circulation (48–50), which likely poses the gland to a greater risk of ischemic harm (**Figure 2**). In particular, long hypophyseal portal vessels substantially represent the only source of vascularization of the lateral portion and of pars tuberalis (mostly populated by GH, PRL and FSH/LH secreting cells) (52) (**Figure 3**). Instead, the antero-medial portion and the central wedge (mostly populated by TSH and ACTH secreting cells) (52) (**Figure 3**) receive a mixed supply by both long and short hypophyseal portal vessels (48–50) (**Figure 2**). Therefore, the ischemic susceptibility hypothesis may be one of the most plausible explanation for the differential frequency of pituitary axes involvement after TBI. In fact, the most vulnerable axes (GH and FSH/LH) are those whose blood supply only relies on long hypophyseal portal vessels, that are by themselves more prone to vascular damage; instead, the most resilient ones (ACTH and TSH) are those whose blood supply is guaranteed both by long and short hypophyseal portal vessels (48–50, 52) (**Figures 2**, **3**).

The third and last major mechanism implied in the pathophysiology of secondary pituitary injury after trauma is inflammation. Part of the inflammation mechanisms affecting the pituitary gland after TBI are probably shared with the general inflammation mechanisms known to affect the brain parenchyma as a whole, with an uncontrolled and self-sustaining release of pro-inflammatory cytokines such as interleukin 1 (IL-1) and tumor necrosis factor α (TNF-α) (53).

In addition, a pituitary-specific inflammatory mechanism may be related to autoimmunity and, more specifically, to the presence of anti-hypothalamus antibodies (AHA) and/or anti-pituitary antibodies (APA). The positivity to these antibodies was demonstrated to be more frequent in patients with previous TBI (ranging from 44% to 60% depending on the study) than in matched healthy controls (0%) (54, 55). A similar result, even if less pronounced, was found in a cohort of subjects who underwent chronic repetitive head trauma due to amateur boxing activity, in which a higher positivity of AHA (21%) and APA (23%) was found compared to matched controls (0%) (56). Moreover, among patients with previous TBI, various authors have demonstrated a statistically significant correlation between the presence of AHA and/or APA positivity and that of post-traumatic hypopituitarism, with an odds-ratio (OR) ranging from 2.2 to 8.5, depending on the study (54–56). Looking at the whole body of these results, it is therefore reasonable to hypothesize that autoimmunity may play a role in the pathogenesis of TBI-induced hypopituitarism. The genesis of AHA and/or APA production is likely related to hypothalamus and/or pituitary exposure and release of antigens that would have otherwise remained unexposed. Despite the aforementioned association, the available evidence is not clearly sufficient to establish whether AHA/APA positivity may actually play an active pathophysiological role in the propagation/perpetuation of TBI-related pituitary damage, or may instead represent only an epiphenomenon of hypothalamic/pituitary antigen exposure due to necrotic post-TBI alterations of these areas.

Finally, another potential pituitary-specific inflammatory modulation mechanism may be related to individual intrinsic genetic susceptibility. In the general context of traumatic brain injury, ApoE polymorphisms have been widely shown to be associated with various clinical outcomes after TBI, such as the onset of seizures (57), coma duration (58), and subsequent neurobehavioral recovery (59). In fact, ApoE is a key protein in enhancing lipid transport and metabolism within the nervous system and has a role in neuronal repair and maintenance. In the specific context of post-traumatic hypopituitarism, a study from Tanriverdi et al. confirmed the possible role of ApoE polymorphism on neuroendocrinological outcomes; in particular, this study demonstrated a likely protective role of ApoE3/E3 genotype against the development of post-traumatic pituitary disfunction, with an estimated OR of 0.29 (60).

### Evidence From Histopathological Studies

Most histopathological studies in patients with TBI-related hypopituitarism have been published several decades ago, as autoptic case series. These studies showed that the most frequent pathological alterations of pituitary gland after TBI were anterior lobe ischemic necrosis, posterior lobe haemorrhage and pituitary stalk haemorrhage, each occurring in approximately 14-42% of patients (61–64). A normal pituitary could be found in a variable proportion of 14-74% of patients, depending on the study (61–64).

More recently, another study by Salehi et al. (65) provided a further insight into histopathological changes of the pituitary gland after TBI. The overall results were not in contrast with previous case series, but a more accurate analysis relating pathological findings to the timing of death after trauma has to be discussed. In fact, the authors showed that no abnormalities of the pituitary gland could be found in patients who died immediately after trauma, both at macroscopic and at microscopic level. On the other hand, several degrees of pituitary haemorrhage and/or ischemic necrosis could be found in 43% of patients who survived between 3 h and 7 days after trauma. Moreover, the extent of histopathological alterations in those patients was significantly related to the time elapsed between injury and death (< 10% of adenohypophysis involved in patients who died within 1 day, > 50% in patients who died between 1 and 7 days) (65).

In conclusion, the whole body of histopathological evidences supports the hypothesis that post-TBI neuroendocrine damage could be mostly related to pituitary vascular vulnerability. However, despite the interest of these results, it must be pointed out that they are based on autoptic studies looking at a very specific and selected subset of TBI patients, i.e. those dying in the acute phase after a severe TBI. This undoubtedly represents an important selection bias, as this category of patients accounts for just a small minority of all patients suffering from TBI. Moreover, this is a category of TBI patients in which correlation with hormonal outcomes is neither feasible nor relevant. Precise pituitary histopathological correlations in long-term TBI survivors are thus lacking, but further research in this field are clearly conditioned by the constitutional limit to conduct this kind of evaluation *in vivo*.

### Evidence From Imaging Studies

Several studies have been published about the possible identification of microstructural abnormalities of the pituitary gland by imaging techniques in patients with TBI-related hypopituitarism. Also in this subset of patients the imaging modality of choice for the evaluation of the pituitary gland was mostly MRI.

No single features were found to predict with high accuracy the presence or the absence of hypopituitarism in patients experiencing TBI. Therefore, in clinical practice the role of imaging in the prediction of post-traumatic hypopituitarism is limited. However, the available evidence is still of significant interest, as it provides information on the possible pathophysiological mechanisms through which post-traumatic hypopituitarism develops.

In this regard, the role of imaging is surely less accurate than that of pathological studies, considering that the deduced evidences about the underlying pathophysiological mechanisms can be only indirect. On the other hand, the clear advantage is that these evidences may be obtained for all patients with TBI, regardless of trauma severity and mortality.

In the acute phase, Maiya et al. (66) showed a significant enlargement of pituitary gland dimensions after TBI compared to healthy controls. In particular, this enlargement was evident not only in those patients with focal changes of the pituitary gland (haemorrhagic foci, heterogeneous signal intensities, macroscopical swelling) but also in those without specific focal pituitary abnormality. This is consistent with a pathophysiological hypothesis of an underlying pituitary edematous change, which is likely to be predominant in the first days after TBI. However, in this study no data about the subsequent pituitary functional outcomes were available, neither in the short nor in the long term. Therefore, it is not possible – based on these data – to explore the potential correlation of pituitary dimension in the acute phase and the subsequent development of pituitary deficits.

In the subacute phase, Zheng et al. (67) showed a reduction in apparent diffusion coefficient (ADC) in diffusion-weighted imaging (DWI) of pituitary gland in patients with previous TBI compared to healthy controls. DWI is based on the capacity of MRI to demonstrate the random, brownian diffusion movement of water. It is widely recognized as one of the best imaging modalities of cerebral ischemia, in which failure of the energy-dependent Na-K-ATPase determines a translocation of water from the interstitial to the intracellular space, and therefore a reduction of its diffusion capacity (68). This diffusion restriction determines a coherent change in signal intensity on DWI and on the derived ADC map, in which ischemic areas appear characteristically hypointense (69, 70). The results obtained by Zheng et al. thus confirm the probable role of ischemia in pituitary microstructural change in the first few weeks after TBI. Interestingly, even if ADC reduction with respect to healthy controls was found to be significant in the whole cohort of TBI patients, this alteration resulted to be more prominent in those which subsequently developed pituitary deficits at the hormonal follow-up; this is a further proof of the plausible relation between the severity of microstructural ischemic abnormalities in the short-term and the final functional outcome in the long-term.

In the chronic phase, Schneider et al. (71) showed that, after TBI, pituitary abnormalities at imaging were more frequent in patients with some degree of hypopituitarism (80%) than in those without pituitary hormone deficits (29%). The most common finding, in particular, was pituitary volume reduction, up to the degree of empty sella in some patients. The pathophysiological process underlying these changes is plausibly related to the long-term sequelae of pituitary tissue necrotization and subsequent fibrosis, which likely contribute to irreversible tissue loss.

The evidence of a pituitary volume reduction in the chronic phase finds further confirmation in a study by Tanriverdi et al. (72), in which the authors examined pituitary function and pituitary volume in a cohort of amateur boxers. As it is known, boxers are exposed during their career to mild chronic repetitive head traumas, which may determine pathophysiological consequences similar to classical TBI in the long term. In this study, 18% of boxers had some degree of pituitary dysfunction, and the presence of one or more hormonal deficits was significantly correlated with a lower pituitary volume at MRI.

## TBI-Related Hypopituitarism: Clinical Presentation

Post-traumatic hypopituitarism (PTHP) can present with variable and non-specific clinical features, which may overlap with those resulting from the injury. The consequence is a delay in diagnosis, with a higher morbidity and mortality (39).

The clinical picture presents a very large spectrum determined by the kind, number and severity of hormonal deficiencies and could go from mild and non-specific complaints to life-threatening conditions (73).

Traditionally, an acute and a chronic phase can be distinguished, but pituitary dysfunction during the acute phase does not necessarily lead to long-term hypopituitarism. In a systematic review, the analysis of prospective studies showed that some of the early abnormalities are transient with complete recovery, while hypopituitarism can evolve over time and become detectable only later (8).

The first 2 weeks after trauma are considered the acute stage.

The most common hormonal changes in this phase are represented by gonadotropin and GH deficiency, but in the clinical presentation they are not the most evident.

In fact, the most fearsome endocrinological complication of the acute stage is adrenal insufficiency. Hyponatremia, hypoglycemia, hypotension, fatigue, mental confusion are some of its typical features. Patients affected by hypocortisolism require a higher dose of vasopressors and have a higher mortality rate; therefore hormone replacement therapy is crucial (74, 75).

Impaired vasopressin secretion (Syndrome of Inappropriate Antidiuretic Hormone Hypersecretion - SIADH or Central Diabetes Insipidus - CDI) could also be life-threatening, contributing to the hydro-electrolytic imbalance of the acute phase (76).

Hypothyroidism can be also reported, due to the adaptive response after trauma and the use of steroids. It contributes to the clinical picture of marked asthenia, lethargy and confusion, not always easy to discriminate in the acute stage.

GHD is considered a common alteration of the acute phase, reflecting the adaptive response after the traumatic event.

Hyperprolactinemia could be reported as a consequence of pituitary stalk compression or as a physiological reaction to stress. Together with hypogonadism, hyperprolactinemia may lead to the menstrual alteration and the sexual dysfunction of the post-TBI period.

Some of these abnormalities are transient: recovery from hypoadrenalism is described in 50% of patients, from CDI in up to 90% (77). Hypothyroidism and SIADH often resolve in 3-12 months (40).

The chronic phase starts at 3 months after TBI. The clinical features could be very variable and not specific, depending on the different axes involved. Lethargy or insomnia, fatigue, reduced attention, difficulty concentrating, memory impairment, anxiety, depression, irritability and diminished libido are often described (78).

Also in this phase GHD and hypogonadism are the most frequent anomalies, hypocortisolism and central hypothyroidism are relatively rare and CDI could persist in a small percentage of patients (79).

These patients experience metabolic abnormalities, neurocognitive impairment, and a decreased quality of life (34, 80).

### Metabolic Alterations

In patients with PTHP, altered function of hypothalamic nuclei and pituitary disfunction itself determine an adverse metabolic profile. Glycemic disorders, dyslipidemia, weight gain with abdominal fat distribution, changes in body composition and reduced bone mineral density determine the higher morbidity and mortality of these patients (78).

Different studies demonstrated that PTHP patients have higher BMI, increased LDL cholesterol and total cholesterol (19), altered glucose levels and insulin resistence (34).

Decreased thyroid function reduces the basal metabolic rate and hypogonadism affects bone and muscle mass, beyond the effect on libido and reproduction. It is however GHD that plays the major role, affecting glycemic and lipidic profile, increasing BMI and waist circumference, reducing bone mineral density and leading also to anemia (78).

GHD can affect also the rehabilitation: patients with post-TBI GHD seem to have lower aerobic capacity, a measure of physical resistance, which may delay or inhibit the recovery process (81).

### Neurocognitive Alterations

Recently, more attention was paid to neuropsychiatric symptoms which are due both to PTHP and brain injury itself. Cognitive and affective impairment may be severe, prejudicing patients’ social skills. In fact, attention, memory, executive functions and language can be involved.

Symptoms of PTHP can overlap with cognitive, sleep, mood, and anxiety disorders due to Post-Traumatic Stress Disorder (PTSD). Differential diagnosis can be challenging, but essential for the therapeutic implications.

Hypothyroidism is associated with neurocognitive function: low speed of information processing and deficits in short-term memory are the most described (73).

Hypogonadism is also associated with cognitive dysfunction: patients with lower testosterone levels after TBI seem to have an increased risk for Alzheimer’s disease (82).

Also hypoadrenalism results in mood disorders, decreased memory, and frank psychosis in the chronic phase, in addition to the classical picture of fatigue, weakness, and impaired response to stress (78).

Although every hypothalamic-pituitary axis could be involved in cognitive functions, the role of GH and the effects of its deficiency are the most known and frequently observed (83).

In fact, somatotropic axis has a role in microtubular regeneration, dendritic growth and regrowth, regulation of the use of glucose in the brain and, probably, an action on hippocampal area. So, GHD may lead to impaired neuronal, somatic, and dendritic growth, affecting memory and cognitive function too (78, 84).

Several studies reported that patients with post-traumatic GHD have more severe cognitive impairment, in particular deficit in attention and memory, as well as emotional problems, than those with normal GH values (33, 85, 86).

Patients with post-TBI GHD have a higher risk to achieve poor cognition outcomes than those with an intact somatotropic axis after trauma (87). Park et al. showed that patients with GHD after TBI have decreased cerebral glucose metabolism in specific cortical areas involved in intellectual function, executive function, and working memory (88).

In contrast to all these studies, Pavlovic et al. did not find significant differences between patients with or without GHD after brain injury (89).

### Quality of Life (QoL)

Another important feature is QoL: patients with post-TBI GHD are more likely to be depressed and with a poorer quality of life than those with GHD due to other causes. In particular, domains of physical health, energy and fatigue, emotional well-being, pain, and general health seem to be affected (85).

Also perceived poor QoL would negatively impact on rehabilitation after TBI.

## TBI-Related Hypopituitarism: Diagnosis

PTHP, and in particular GHD, are often underdiagnosed: patients with post-TBI GHD seem to be diagnosed on average two and a half years later after the primary onset of disease when compared to those with Non-Functioning Pituitary Adenoma (NFPA) (87).

PTHP diagnosis is not different from hypopituitarism due to other causes. However, the time and type of hormonal assessment in TBI patients is controversial.

### Who to Test

Despite the severity of trauma itself, hypopituitarism can develop in patients post-TBI, but testing all of them is not feasible because of the great amount of human and economic resources needed. A rational approach is to evaluate life expectancy, avoiding to test patients with poor prognosis who cannot benefit of hormonal replacement therapy. Conversely, patients with mild TBI could develop hypopituitarism, but not all of them achieve medical attention (90) and testing is not considered cost-effective (87).

Epidemiology could be useful to establish who to screen: in fact, PTHP frequency is better established in patients with moderate or severe TBI based on GCS score. Furthermore, other risk factors include age, intracranial hemorrhage, focal cortical contusion, seizures and skull base fracture.

In patients symptomatic for acute hypopituitarism (i.e. electrolyte unbalance and/or acute adrenal insufficiency) an endocrinological evaluation is mandatory.

Patients with mild TBI who need hospitalization (more than 24 h), a neurosurgical intervention, monitoring in an Intensive Care Unit, or those who present anatomical changes on CT scan, are considered complicated and screening is also recommended (87). Conversely, patients with a mild uncomplicated TBI should be screened for hypopituitarism only if the clinical suspect is present (91).

Patients who required hospitalization for at least 24 h, those with radiological abnormalities on CT scan, and those who presents signs and symptoms of PTHP should be screened at three months and one year post-TBI. It is possible to perform hormonal screening even further, if symptoms persist (78).

### Biochemical Evaluation: Time and Kind

Pituitary function evaluation could be challenging in the acute phase post-TBI. In fact, in this stage patients have hormonal changes as part of the stress response and acute adaptive response to injury. Hormonal levels are also affected by medications and surgery. Pituitary function during this time could be variable (78).

While some of the alterations are natural consequences of the trauma itself, others are life-threatening and require an immediate hormonal replacement therapy. So, basal hormonal evaluation should be performed in any patient with TBI who has been hospitalized and presents signs and symptoms suggesting adrenal insufficiency (92). For the diagnosis, a morning serum cortisol should be measured. Usually, a level beyond 15 µg/dL indicates a proper function, values between 3 and 15 µg/dL require further investigation using a stimulation test, while a concentration lower than 3 µg/dL allows a diagnosis of hypoadrenalism (93). In the post-TBI context, these cut-points are not considered applicable. In fact, plasma cortisol concentrations rise in parallel to the severity of acute illness, so Hannon et al. proposed that critically sick patients should have plasma cortisol levels >300 nmol/L (=10.875 µg/dL), suggesting the need of hormonal replacement therapy for lower values (74). Also the evaluation of cortisol reserve with a dynamic test seems to be not reliable in TBI patients, at least in the acute phase. In fact, Endocrine Society Clinical Practice Guidelines suggest performing an ACTH stimulation test (Synacthen test) when required for secondary adrenal deficiency diagnosis (93), preferring the low-dose (1 µg) version to reduce false negative results due to a supraphysiological stimulus (94, 95). However, in the setting of the acute phase of TBI a Synacthen test is inappropriate because of the acute nature of ACTH deficiency, that would not have time to lead to secondary adrenal insufficiency. Therefore, patients with TBI would have a normal response also to the low-dose ACTH stimulation test (74). Even an extremely accurate test as Insulin Tolerance Test (ITT) is not feasible for safety concerns in patients with TBI in the acute phase.

First post-acute phase evaluation should be scheduled 3-6 months after TBI. Thyroid and gonadal axes are frequently involved, so they should be evaluated. TSH and fT4 are dosed to confirm or exclude hypothyroidism, while gonadic evaluation is different between males and females. In men with suspected hypogonadism LH, FSH, testosterone and PRL levels should be dosed. In women of reproductive age with menstrual irregularities is recommended to measure PRL, LH, FSH and estradiol (E2), while in postmenopausal women gonadotropins reduction may be sufficient for the diagnosis (78, 83, 93). The adrenal axis should be re-evaluated measuring morning serum cortisol and, eventually, using an appropriate stimulation test.

To assess for CDI in patients with polyuria, serum and urine osmolality should be dosed simultaneously: urine osmolality/plasma osmolality ratio should be ≥2, after excluding glycosuria (78).

Somatotropic axis evaluation is recommended at least six months after TBI because of the possible spontaneous recovery in the post-acute phase (87). Some Authors proposed to postpone the evaluation until 1 year after TBI in adults, while children may require an earlier assessment (83). The diagnosis of GHD could not be based only on IGF-I levels; even if in patients with low IGF-I levels and multihormonal deficiency GHD diagnosis does not require further tests (96), in all other conditions different dynamic tests are available to confirm the suspected somatotropic deficiency with a high accuracy (96, 97). The choice of a test over another is usually based on patient’s characteristics, availability of secretagogues and center experience (87). ITT is considered the gold standard and it evaluates both hypothalamic and pituitary integrity, but it can be used only after excluding contraindications (i.e. seizure, cardiac disease) (8, 90). Up to 22% of patients who had TBI develop seizure disorders, so ITT is often considered not safe in this setting (98).

The administration of GH Releasing Hormone (GHRH) plus Arginine (Arg) or GHRH plus GH Releasing Peptide 6 (GHRP-6) provides a strong stimulus to GH secretion and are considered safe, so they could be used as a dynamic test for GHD evaluation. However, they are not useful for GHD of hypothalamic origin and both GHRH and GHRP-6 are unavailable in many Countries (96). Glucagon Stimulation Test (GST) is not usually the first choice for GHD diagnosis. In fact, it could be labored, requiring a monitoring of at least 3 h, and it could lead to delayed hypoglycaemia (96). The diagnostic accuracy could be lower in patients with glucose intolerance (97).

For all GHRH+Arg, GHRH+GHRP-6 and GST different BMI-related cut-off points are available (99–101). Also for ITT new BMI-related cut-points have been recently proposed, allowing to avoid false positive results due to obesity (102). However, they need further validation.

Lately, Macimorelin test has been proposed for the diagnosis of adult GHD (97), but data about its use in post-TBI patients are lacking until now.

An overview of the main stimulation tests for GHD diagnosis is provided in **Table 1**.

**Table 1:** Main characteristics of GH stimulation tests.

Test	ITT	GHRH+Arg	GHRH+GHRP-6	GST	Macimorelin
**Drug and dose administered**	Human Regular Insulin 0.1-0.15 UI/kg iv.	GHRH 1-44 1 µg/kg iv + Arginine HCl 0.5 g/kg (max 30 g) infusion.	GHRH 1-44 1 µg/kg iv + GHRP-6 1 µg/kg iv	Glucagon 1-1.5 mg im.	Macimorelin 0.5 mg/kg in 1 ml/kg of water oa.
**Sampling and measurements**	GH and glucose at times 0′-30′-45′-60′-90′	GH at times 30′-45′-60′	GH at times 0′-15′-30′	GH and glucose at times 0′-30′-60′-90′-120′-150′-180′-210′-240′	GH at times 30′-45′-60′-90′
**GH cut-points (µg/L)**	- <5 (partial) or <3 (severe). - ≤3.5 if BMI <25 kg/m^2^; (*) - ≤1.3 if BMI 25-30 kg/m^2^; (*) - ≤2.2 if BMI >30 kg/m^2^. (*)	- ≤11.5 if BMI <25 kg/m^2^; - ≤8 if BMI 25-30 kg/m^2^; - ≤4.2 if BMI >30 kg/m^2^.	- <10 if BMI ≤35 kg/m^2^; - <5 if BMI >35 kg/m^2^.	- <3 if BMI <25 kg/m^2^; - <1 if BMI ≥25 kg/m^2^.	≤2.8
**Side Effects**	- Severe hypoglycaemia; - Late hypoglycaemia.	Flushing, nausea, smell and taste disorders	Flushing	Delayed hypoglycaemia, nausea, vomiting	Dysgeusia
**Contraindications**	Pregnancy, older age, history of seizure, history of CAD.	Chronic renal failure.	None	Severe fasting hyperglycaemia.	Use of drug that prolong QT.
**Pros**	- Possible simultaneous assessment of HPA function; - Evaluation of both hypothalamic and pituitary integrity.	- Strong selective stimulus; - Safe test.	- Strong selective stimulus; - Safe test.	Evaluation of both hypothalamic and pituitary integrity	- Oral administration; - High tolerability.
**Cons**	Symptomatic hypoglycaemia (<40 mg/dl) not always achieved in diabetic patients and obese.	- Not useful for GHD of hypothalamic origin; - GHRH not commercially available in every Country.	- Not useful for GHD of hypothalamic origin; - GHRH and GHRP-6 not commercially available in every Country.	- Long time needed; - Labored test; - Lower accuracy in patients with glucose intolerance.	Expensive
**Notes**	Gold standard for GHD diagnosis; (*) Need further validation of BMI-dependent cut points.	///	///	Not frequently the first choice for GHD diagnosis	Safety and diagnostic performance not available for patients <18 and >65 years.

ITT, Insulin Tolerance Test; GHRH, Growth Hormone-Releasing Hormone; Arg, Arginine; GST, Glucagon Stimulation Test; iv, intravenous; im, intramuscular; oa, oral administration; GHD, Growth Hormone Deficiency; BMI, Body Mass Index; CAD, Coronary Artery Disease; HPA, Hypothalamus-Pituitary-Adrenal.

## TBI-Related GHD: Management and Outcomes

### Rationale for Treatment of Post-Traumatic GHD

GH is expressed not only at pituitary level but also in many other organs and tissues, including the central nervous system (CNS) (103) where it plays an important role in the regulation of cell proliferation and survival (104–107). It is well known that GH plays a role in brain repair, and several preclinical and clinical studies have demonstrated the positive effects of GH treatment on neurogenesis in both animals and humans (108, 109). Moreover, the whole GH system [GH, GH receptor (GHR) and IGF-I] is acutely and strongly upregulated after brain injury (110, 111), and specifically associated with stressed neurons and glia (112–114). However, to date, the role exerted by GH at CNS level and, in particular, its possible contribution to the recovery of neurologic injuries remains poorly understood.

GH exerts its beneficial effects on neural repair through different mechanisms that include regulation of the proliferation, survival, differentiation and migration of both neural progenitors and newly formed neurons.

Both *in vitro* and *in vivo* studies support the ability of GH to promote the proliferation of neural precursor. GH treatment promotes proliferation of both human fetal (115) and adult mice neural stem cells (NSCs) (106, 116). It has been demonstrated that peripheral administration of GH is able to induce cell proliferation in the brain of both normal (117) and hypophysectomized (107) adult rats (117); moreover, peripheral administration of GH seems to increase the proliferative response of hippocampal progenitors to kainate-induced injury (118). Despite the main role of GH on the proliferation of neural precursors, it has been suggested that GH may actually have a more prominent effect in the regulation of survival, differentiation, or even migration of newly formed neurons (113). In agreement with the control exerted by GH on cell survival, GH prevents the apoptotic death of both mature neurons (119–121) and primary neurospheres derived from embryonic mouse NSCs (122). On the other hand, an increased apoptotic death of NSCs is observed during the treatment with a GHR antagonist (123). Lastly, GHD has been shown to impair the survival of newborn neurons in the subgranular zone of adult rat dentate gyrus (124), while elevated GH levels within the hippocampus reduce apoptosis (125). All these neuroprotective effects would explain some of the acute consequences of GH therapy. Several studies support a role for GH in enhancing neuronal precursors differentiation (126, 127), while a possible effect of GH on neuronal precursors migration is less clearly established.

Finally, it cannot be ruled out that GH promotes neurogenesis and neurorepair, at least in part, through indirect mechanisms including both the synthesis and the release of IGF-I, epidermal growth factor (EGF) or erythropoietin (EPO) or changes in neurotransmitter turnover.

IGF-I is the main mediator of GH action and it is essential for CNS development (128, 129). IGF-I stimulates the proliferation of neuronal precursors and the survival and differentiation of both neurons and oligodendrocytes (130). As a result, brain growth is increased by IGF-I overexpression and reduced as a consequence of decreased IGF-I levels.

In this context, it must be pointed out that IGF-I has been shown to be a crucial modulator of CNS activity, including higher functions like cognition, and to modulate genes involved in microvascular structure and performance, and synaptic plasticity (131, 132).

On the other side, EGF has been demonstrated to be a powerful mitogen capable of inducing neurogenesis both in basal studies and after experimental injuries (133, 134). Since it has been shown that GH is able to induce the expression of both EGF and its receptor (EGFR) and to mediate EGFR activation (135, 136), it cannot be excluded that the action performed by GH at CNS level is mediated by these interactions.

EPO and its receptor (EPOR) are other factors involved in neurogenesis. Both EPO and EPOR have been identified in numerous areas of the CNS during development, and they are expressed in several neuronal cells like neurons, astrocytes, oligodendrocytes, microglia and cerebral endothelial cells, where EPO activates anti-apoptotic, anti-oxidant and anti-inflammatory signals and stimulates angiogenesis and neurogenesis. It is therefore not surprising that EPO can determine a strong protective effect on neuronal tissue in experimental models of stroke, cerebral hemorrhage, traumatic brain injury, and neuroinflammatory and neurodegenerative diseases (137).

Moreover, it has been shown that the blockade of EPOR in the CNS leads to impairments in neural cell proliferation and survival during embryonic development, and in post-stroke neurogenesis in adult brain, further confirming EPO’s role in neurogenesis (138, 139). Finally, both *in vivo* and *in vitro* studies show that EPO is able to increase oligodendrogenesis and remyelination after stroke (140). Since GH induces EPO release from kidneys (141), it can be assumed that GH promotes neurogenesis and neurorepair, at least in part, through indirect mechanisms including EPO mediated effects.

Peripherally, GH is an anabolic hormone that promotes growth in skeletal and soft tissues (142) through the expression of GHR at many levels including liver, muscle, bone, and adipose tissue (143). GH plays an important role in metabolism stimulating lipolysis, reducing hepatic triglyceride secretion, activating the nitric oxide system (and so reducing vascular tone), increasing cardiac performance and exercise capacity, and promoting longitudinal skeletal growth (142). In patients with traditional causes of hypopituitarism such as pituitary tumors, GHD is associated with changes in body composition, worrisome metabolic dysfunction, reduced bone density, a significant decrease in QoL, increased cardiovascular risk, and impaired cardiac function (33, 144). GH replacement therapy has been shown to be partially beneficial in adults with GHD (145). However, the evidence of the benefit from GH replacement therapy in post-traumatic GHD is scant: more robust data are available about improving cognition and QoL, but not about all the other parameters.

Literature data on the possible beneficial effects of recombinant human GH (rhGH) therapy in patients with GHD post-TBI are currently scarce and fragmented. The studies available so far are few (14, 146–153), carried out on small series (147–150, 152–154), extremely variable with respect to the type of patients considered (severity of TBI, age and sex of the subjects, presence or absence of other associated hormonal deficits, time elapsed since TBI), the type of control group (subjects with GHD from another cause, subjects with GHD from TBI, subjects with previous TBI but without GHD), the way in which the condition of GHD is defined (different stimulation tests, some of which not even recognized for GHD diagnosis by the international guidelines), the rhGH dose which in some cases appears supraphysiological (148, 150), the outcomes considered and the tools used for their detection. Furthermore, the studies in general have a short duration (3-12 months) and the drop-out is often high (153). All these factors make the results of every single study difficult to compare and the results of every individual experience on a broader case series are scarcely generalizable.

An overview of the main studies investigating GH replacement therapy in adult post-TBI GHD patients is provided in **Table 2**.

**Table 2:** Studies investigating GH replacement therapy in adult post-TBI GHD patients.

Authors	Study design	GHD post TBI patients (N)	Control group (N)	Age of treated patients	GHD testing	rhGH dose^§^	Duration of rhGH treatment	Time elapsed from TBI	Parameters analyzed
Kreitschmann-Andermahr I. et al. (146)	Retrospective database analysis	84	84 GHD patients due to NFPA	Patients: 36.7 ± 10.8 yrs Controls: 37.3 ± 10.3 yrs *p = NS*	ITT (31 TBI+39 NFPA); GHRH+Arg (4 TBI+8 NFPA); GHRH (16 TBI+18 NFPA); Arg (49 TBI+31 NFPA)	TBI: 0.41 ± 0.3 mg/day NFPA: 0.36 ± 0.2 mg/day	12 months*	CO-GHD: 21.0 ± 8.1 yrs AO-GHD: 6.2 ± 9.4 yrs *p = 0.000*	BMI; WHR; IGF-I SDS; GH-dose; fasting lipid profile; QoL-AGHDA
High W.M. et al. (14)	Open, prospective, randomized study	12 (5 GHD+7 GHI^#^)	11 TBI (3 GHD+8 GHI)	Patients: 36.1 ± 10 yrs Controls: 39.1 ± 8.5 yrs *p = NS*	GST	Patients: 0.6 mg/day or uptitrated to achieve an IGF-I level in the upper half of the normal range Controls: *placebo*	12 months	Patients: 1.8-33.9 yrs Controls: 1.9-13.8 yrs *p < 0.058*	Muscle biopsy; VO_2_; muscle strength; LBM; FM; language; visual/spatial functioning; upper extremity motor functioning; information processing efficiency; working memory/attention; learning and memory; executive functioning; intellectual functioning; emotional functioning
Maric N.P. et al0 (147)	Open, prospective study	4	2 GHD post TBI	39.3 ± 11 yrs	GHRH+GHRP-6	M: 0.3 mg/day; F: 0.4 mg/day	6 months	≥ 3 yrs	Psychiatric assessment: Zung Depression Inventory and SCL-90-R. Neuropsychological examination: MMSE, RAVLT, RCF, TMT, BNT, WCST
Reimunde P. et al. (148)	Open, prospective, placebo-controlled study	11	8 TBI without GHD	Patients: 53.4 ± 17.4 yrs Controls: 47.1 ± 14.6 yrs	GHRH+Arg	Patients: 1 mg/5 days/week Controls: *placebo* (All subjects received daily cognitive rehabilitation)	3 months	Patients: 44.6 ± 35.6 yrs Controls: 46.6 ± 28.8 yrs	Neuropsychological test battery (WAIS)
Moreau O. K. et al. (149)	Open, prospective, controlled study	23	27 TBI (15 without GHD + 9 with partial GHD° + 3 GHD who refused rhGH)	Patients: 37.9 ± 11.7 yrs Controls: 37.1 ± 12.4 yrs *p = NS*	GHRH+Arg ITT	0.2-0.6 mg/day	12 months	Patients: 7.8 ± 6.6 yrs Controls: 5.5 ± 6.2 yrs	BMI; Health-related QoL (QOLBI); RCF; TAP; NRS-R; pADL; iADL
Devesa J. et al. (150)	Open, prospective study	5	8 TBI without GHD	Patients: 27.4 ± 4.8 yrs Controls: 26.3 ± 12.9 yrs	GHRH+Arg	Patients: 1.0 ± 0.0 mg/5 days/week resting 15 days every 2 months Controls: 0.94 ± 0.1 mg/5 days/week resting 15 days every 2 months (All subjects received daily rehabilitation according to the specific individual needs)	Patients: 11.2 ± 1.6 months Controls: 8.9 ± 2.2 months	Patients: 44.1 ± 34.3 months Controls: 66.8 ± 47.2 months	Cognitive assessment (WAIS, MMSE); motor assessment (FAC, Tinetti); swallowing function (FOAMS); visual function; functional assessment (MBI); IGF-I
Gardner C. J. et al. (151)	Retrospective database analysis	161	1268 GHD patients due to NFPA	Patients: 42.6 yrs [40.8; 44.5 yrs] Controls: 53.2 yrs [52.5; 53.8 yrs] p < 0.0001	IGF-I GHRH+Arg Arg GST GHRH ITT Other	Patients: 0.37 [0.35; 0.40] mg/day Controls: 0.33 [0.32; 0.34] mg/day p = 0.006	12 months	Not available	BMI; WHR; IGF-I SDS; LBM; FM; BP; glucose metabolism; GH-dose; fasting lipid profile;, QoL-AGHDA
Leonhardt M. et al. (152)	Open, prospective study	4 patients with isolated post-BI GHD (only 1 post TBI)	6 TBI patients without hormonal deficiency	Patients: 49.0 ± 9.8 yrs Controls: 49.5 ± 13.6 yrs	GHRH+Arg	0.2-0.5 mg/day	6 months	Patients: 25-1024 days Control: 41-2566 days	QoL (SF-12; EQ-5D; QoLBI; BDI; PSQI); Cognition (VLMT; test from the psychological TAP 2.3 test battery of attention); BMI; Abdominal fat distribution
Dubiel R. et al. (153)	Randomized, prospective, placebo-controlled study	31^	32 TBI^	Patients: 32.2 ± 15.2 yrs Controls: 30.1 ± 13.7 yrs p = NS	Arg^	Patients: 0.4 mg/day up- or down-titrated to achieve an IGF-I level in the upper quintile of the range for age and body weight, to a maximum dose of 1 mg/day Controls: *placebo*	12 months^▪^	Patients: 65.7 ± 30.4 days Controls: 62.5 ± 41.4 days *p = NS*	Glucose metabolism; fasting lipid profile; free T4; IGF-I; AEs; GOS-E; DRS; FIM; QoL (SWLS and SF-36); neuropsychological battery

AEs, adverse event; Arg, arginine: AO-GHD, adulthood onset GHD; BDI, Beck Depression Inventory; BI, brain injury (TBI, aneurysmal subarachoid hemorrhage, ischaemic stroke); BMI, body mass index; BNT, Boston Naming Test; CO-GHD, childhood onset GHD; DRS, Disability Rating Scale; EQ-5D, EuroQoL; F, females; FAC, Functional Ambulatory Category; FIM, Functional Independence Measure; FM, fat mass; FOAMS, Functional Outcome Assessment Measure of Swallowing; GHD, Growth hormone deficiency; GHI, Growth hormone insufficiency; GHRH, Growth Hormone Releasing Hormone; GHRH+Arg, Growth Hormone Releasing Hormone+arginine; GHRH+GHRP-6, Growth Hormone Releasing Hormone+Growth Hormone Releasing Peptide-6; GOS-E, Glasgow Outcome Scale-Extended; GST, glucagon test; iADL, independence in instrumental activities of daily living; IGF-I SDS, IGF-I standard deviation score; ITT, insulin tolerance test; LBM, lean body mass; M, males; MBI, Modified Barthel Index; MMSE, Mini Mental State Examination; NFPA, non-functioning pituitary adenoma; NRS-R, Neurobehavioral Rating Scale-Revised; pADL, independence in personal activities of daily living; PSQI, Pittsburgh Sleep Quality Index; QoL, quality of life; QoL-AGHDA, Quality of Life-Assessment of Growth Hormone Deficiency in Adults; QOLBI, Quality of Life after Brain Injury; RAVLT, Rey Auditory-Verbal Learning Test; RCF, Rey-Osterrieth Complex Figure Test; rhGH, recombinant human GH; SCL-90-R, Symptom-check-list; SF-12, 12-Item Short Form Health Survey; SF-36, Short-Form 36; SWLS, Satisfaction with Life Scale; TAP, Test for Attentional Performance; TBI, traumatic brain injury; Tinetti, balance and gait tests; TMT, Trail Making Test; VLMT, Verbal Learning and Memory Test; VO_2_, peak oxygen consumption; WAIS, Wechsler Adults Intelligence Scale; WCST, Wisconsin Card Sorting Test; WHR, waist-hip ratio; yrs, years.

*Data available only from 61 out of 84 TBI patients.

^#^GHI defined as a GH response to GST greater than 3 ng/ml but less than 8 ng/ml.

^§^After titration period.

°partial GHD defined as GH peak > 3 ng/ml but < 10 ng/ml to ITT or > 4.0 to 15.6 ng/ml (depending on the patient’s age and BMI) but < the percentile threshold values from ref. (155) in the GHRH+Arg test.

^Not clear if all patients and controls were GHD subjects: only 24 out of 63 subjects underwent GH stimulation test.

^▪^Only 16 out of 31 patients and 18 out of 32 controls completed 12 months follow-up.

### Evidence for Treatment of Post-Traumatic GHD: Cognition

Several studies reported that patients with post-traumatic GHD have a severe cognitive impairment mainly characterized by deficit in attention and memory, as well as emotional problems, and poor verbal learning (7, 33, 85, 86). GHD also seems to be associated with poor mental health outcomes. The cognition and quality of life problems experienced by patients with GHD may be explained by the reduced expression of GH activity in specific CNS areas involved in memory, learning, and emotions, like the hippocampus and the limbic system (156–158). Park et al. showed that patients with GHD after TBI have decreased cerebral glucose metabolism in specific cortical areas involved in intellectual function, executive function, and working memory (88).

It has been demonstrated that in TBI patients the GH response to stimulation test was negatively correlated to paranoid ideation and somatization (33). GHD patients have a moderate to large impairment in each of the cognitive domains assessed when compared to the matched controls (159) and patients with post-TBI GHD have a higher risk to achieve poor cognition outcomes than those with an intact somatotropic axis after trauma (87). In contrast to all these studies, Pavlovic et al. did not find significant differences between patients with or without GHD after brain injury (89). It is well known that GH replacement therapy in patients with GHD from non-traumatic causes is of benefit (96, 160). A meta-analysis conducted on patients with GHD from any cause showed a moderate improvement in cognitive performance during treatment with rhGH; the improvement concerned mainly memory and attention domains (159).

A beneficial effect of GH replacement therapy on cognition has also been reported in post-TBI GHD patients (14, 148, 150). However, till now, there is not sufficient evidence to support GH treatment with the aim of improving cognition in post-TBI GHD patients.

Recovery during a thorough rehabilitation program after TBI may be positively influenced by normal GH secretion as suggested by Bondanelli et al. who showed that GH peak during GHRH + ARG test was an independent predictor of positive outcomes suggesting that GH replacement therapy may be considered in post-traumatic GHD patients (161). The improvement in cognitive function is more significant and appears earlier than that in motor function; this observation supports the usefulness of GH replacement therapy in the rehabilitation process of these kind of disabilities (159, 162–164). In general, the improvement during GH therapy appears to be more marked in patients with worse cognitive impairment at baseline (149) and it has also been shown that discontinuation of GH therapy is accompanied by a worsening of cognitive performance (147).

Although to date the exact mechanism underlying these effects is not well understood, it cannot be excluded that it could be based on GH stimulating effect on neurogenesis in CNS areas related to recent memory like the hippocampal dentate gyrus. However, stimulation of neurogenesis does not appear to be the mechanism by which GH therapy improves other cognitive processes such as attention and concentration. In this case it has been hypothesized that the effect of GH replacement therapy is mediated by the action of the hormone on some neurotransmitter pathways and this might explain the early responses observed after starting a treatment with rhGH (116, 118, 148, 150).

### Evidence for Treatment of Post-Traumatic GHD: Metabolic and Cardiovascular Risk Factors

Regardless of pathogenesis, GHD is associated with adverse effects on body composition, alterations in glucose and lipids metabolism, reduced physical and cardiovascular performance (165); all these alterations are thought to be the cause of the incresed cardiovascular mortality observed in hypopituitaric patients (166–168). A systematic review showed a small but significant improvement on lean and fat body mass, low-density lipoprotein-(LDL) and total cholesterol, and diastolic blood pressure in GHD adults during GH replacemente therapy (169). In contrast, plasma glucose and insulin levels were increased during GH therapy (169). However, improvement in hard outcomes, such as reductions in cardiovascular events and mortality, has yet to be demonstrated (96, 170).

Several studies argue metabolic disorders in patients with post-traumatic GHD. In patients with post-TBI hypopituitarism, mainly GHD, high LDL and total cholesterol levels, waist circumference, and total fat mass have been showed (19). Treatment of these patients has shown variable results. In one observational study, weight or waist to hip ratio did not change in post-traumatic GHD patients treated with rhGH for a year (146), while in another study there was an improvement in blood pressure, total and LDL cholesterol after 1-year treatment in patients with GHD post TBI (151). A case study of two patients with GHD secondary to sport-related TBI showed some improvement in lipid profile and body composition after a 6-month treatment with rhGH (171).

Patients with TBI have been found to have a reduced aerobic capacity, a well-established measure of physical endurance and fatigue resistance, which may further delay or hinder the rehabilitative process (81). In particular, patients with TBI and a normal GH axis show suboptimal aerobic capacity and those with GHD performed even worse (81). Although GH treatment seems to improve skeletal muscle mass in patients with GHD from non-traumatic causes, data available are not so convincing in post-traumatic GHD patients and the improvement, if any, seems to concern only male patients with TBI and GHD (172). A case study of one patient showed an improvement in muscle force production, body composition, and aerobic capacity after treatment with rhGH for 12 months (173).

### Evidence for Treatment of Post-Traumatic GHD: Bone Health

A higher risk of osteopenia, osteoporosis, and vertebral fractures is observed in hypopituitaric patients adequately replaced with glucocorticoids and thyroid hormones (174) suggesting a fundamental role of both GH and gonadotropins secretion disturbances in the pathogenesis of the reduced bone health observed in these patients. Apparently, in GHD patients there is no difference related to gender and age. GH replacement therapy increases bone mineral density (BMD) (175, 176) and seems to mitigate the increased fracture risk observed in GHD patients (174), but specific data for skeletal outcomes in TBI induced GHD are lacking.

### Evidence for Treatment of Post-Traumatic GHD: QoL

Regardless of the cause, GH is characterized by a compromised QoL (177, 178). Patients with post-traumatic GHD are more likely to be depressed and with a poorer QoL than those with GHD due to other causes (85, 160). Interestingly, patients with post-TBI GHD, when compared to those with GHD secondary to NFPA, seemed to have a less severe GHD at biochemical level, but worse QoL scores (151). In particular, domains of physical health, energy and fatigue, emotional well-being, pain, and general health seem to be affected (85). This perceived poor QoL would negatively impact on rehabilitation after TBI. In general, the treatment of GHD of any cause seems to improve QoL as measured by the QoL-AGHDA (Quality of Life Assessment of Growth Hormone Deficiency in Adults) score and other evaluation tools (179). The QoL improvement is higher in post-traumatic GHD patients than in patients with GHD due to NFPA, especially in the domains of socialization, self-confidence, and tenseness (151). The observed improvement was maintained for a long time, up to eight years, and always based on continuation of treatment.

## Conclusions

TBI is one of the leading causes of disability and mortality affecting many people each year and resulting in a serious burden of devastating health consequences. TBI may lead to transient or permanent pituitary insufficiency. After the initial primary injury, secondary mechanisms that involve an interplay of ischemia, inflammation, and cytotoxicity seem to result in hypopituitarism that causes adverse changes in body composition, worrisome metabolic dysfunction, decreased bone density, and a significant reduced QoL. GHD, the most common pituitary hormone deficiency after TBI, is associated with adverse sequelae, which may impair recovery and rehabilitation. The poor outcomes that are seen with long standing GHD in post TBI patients could be improved by treatment with rhGH, but literature data on the possible beneficial effects of GH replacement therapy in post-TBI GHD patients are currently scarce and fragmented. More studies are needed to further characterize the post-TBI GHD syndrome with the purpose of establishing appropriate standards of care.

## Author Contributions

VG, VC, and FB performed a literature search, wrote the first draft, and designed tables and figures. EG and SG supervised the work and revised the manuscript. All authors contributed to the article and approved the submitted version.

## Conflict of Interest

The authors declare the absence of any commercial or financial relationship that could be construed as a potential conflict of interest.
